# Complex Visual and Auditory Hallucinations Following Neurosurgical Injury: A Case Series and Systematic Review

**DOI:** 10.7759/cureus.94119

**Published:** 2025-10-08

**Authors:** Sophie Jia Qian Koh, Jia Xu Lim, Julian Han, Tien Meng Cheong, David SK Mak, Min Wei Chen

**Affiliations:** 1 Neurosurgery, National Neuroscience Institute, Singapore, SGP

**Keywords:** auditory hallucinations, hallucinations, neurosurgery, perceptual disturbances, visual hallucinations

## Abstract

Hallucinations - perceptions without external stimuli - are complex neuropsychiatric phenomena that remain poorly understood in neurosurgical contexts.

We present two cases of multimodal visual and auditory hallucinations following neurosurgical interventions: a 34-year-old woman with occipital venous stasis following torcular tumor resection and sinus thrombosis, and a 53-year-old man with parieto-occipital edema from a traumatic contusion. Both cases featured vivid, meaningful perceptions without epileptiform activity on electroencephalogram (EEG), suggesting network-level dysfunction rather than focal pathology.

A systematic review of 16 prior non-epileptic cases revealed that visual hallucinations predominated, typically associated with parieto-occipital involvement, while auditory hallucinations were less common and linked to temporal or insular lesions. Notably, concurrent visual and auditory hallucinations, as observed here, have not been previously reported post-neurosurgery. Treatment targeting underlying causes (anticoagulation for venous thrombosis and edema reduction) led to complete symptom resolution within weeks to months, highlighting the importance of etiology-specific management.

These findings demonstrate that hallucinations in neurosurgical patients may result from transient disturbances to association cortices and white matter tracts, with a favorable prognosis when secondary to reversible conditions. The study underscores the need for increased clinical awareness, systematic evaluation to exclude seizures, and tailored therapeutic approaches for this underrecognized complication.

## Introduction

Hallucinations are defined as the perception of an object or event through any of the five senses, without an external stimulus [1]. They represent complex neuropsychiatric experiences that can be distressing for individuals, impairing daily functioning and mental health. These phenomena exist across a sensory and complexity spectrum. Visual hallucinations range from simple, unformed photopsias - which are visualizations of basic shapes, patterns, or flashes of light and color - to complex hallucinations involving meaningful perceptions, such as people, faces, animals, objects, or events. Auditory hallucinations vary from elementary sounds, such as ringing, buzzing, or humming, to meaningful, organized perceptions, such as voices, speech, or music.

The pathophysiology of hallucinations remains poorly understood, though early theories categorized them into psychophysiologic (dysfunction of brain structure), psychobiochemical (dysfunction of neurotransmitters), and psychodynamic (emergence from unconsciousness) [2]. More recent research suggests a more nuanced understanding, with links to psychiatric, ophthalmological, and neurological disorders - such as psychosis, drug-related side effects, migraines, seizures, strokes, and ophthalmic and retinal diseases [3]. To date, there have been 16 neurosurgery-related cases reported in the literature. Although less commonly observed in neurosurgery, iatrogenic visual and auditory hallucinations merit attention, given their debilitating nature.

The authors report two unusual neurosurgical cases of complex, formed visual and auditory hallucinations, review similar neurosurgical etiologies in the literature, and assess their relevance to our findings.

## Case presentation

Case 1

**Figure 1:**
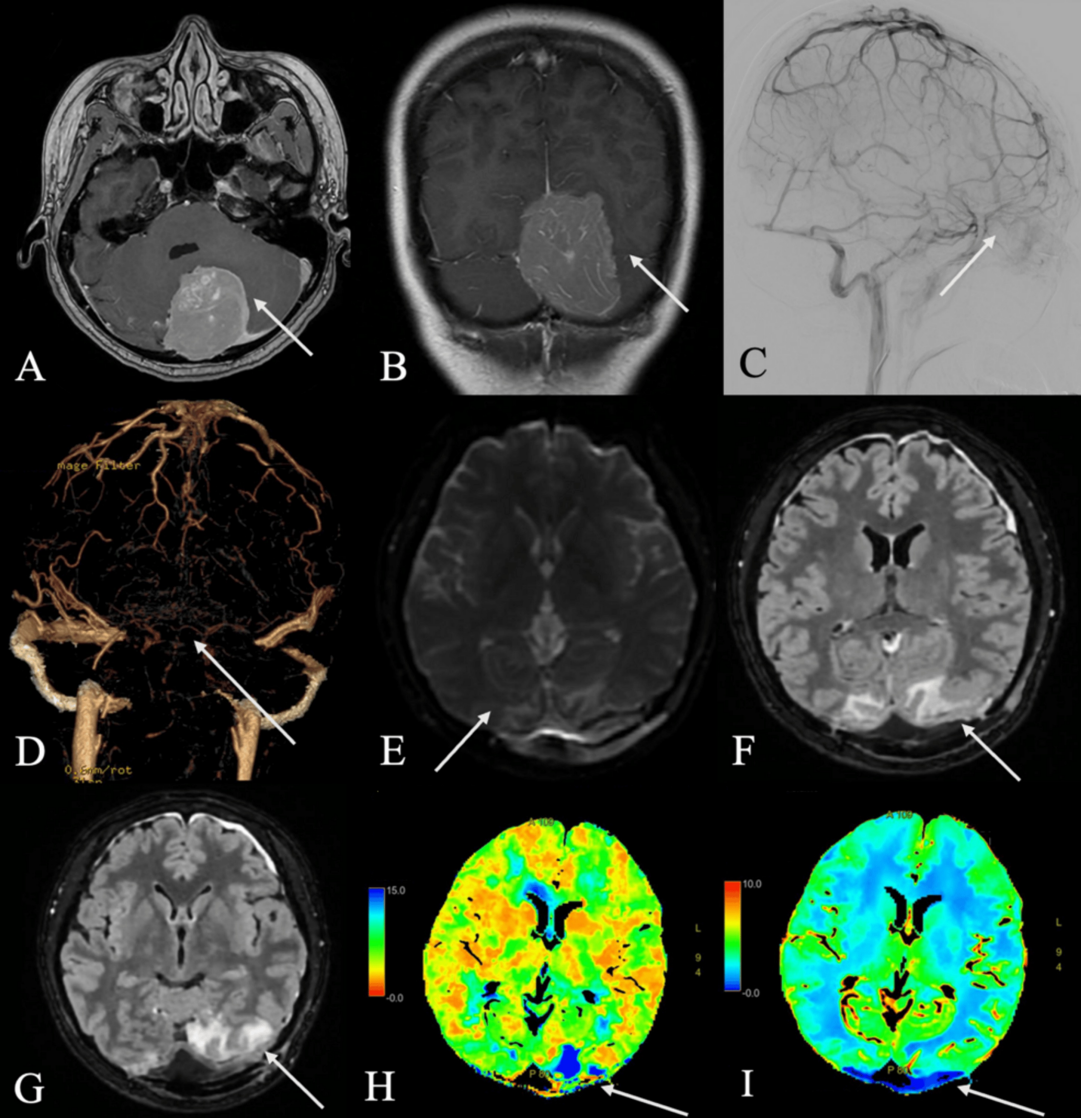
Key Images for Patient 1 Preoperative investigations characterizing lesion: (A) Axial post-contrast T1-weighted MRI demonstrating a large left torcular mass; (B) Coronal post-contrast T1-weighted MRI demonstrating supra- and infratentorial extension; (C) Cerebral angiogram demonstrating absence of opacification of the torcula, posterior SSS, and bilateral TS. Postoperative investigations: (D) CT venogram reconstruction demonstrating filling defect and thrombosis of the straight sinus; (E) Axial diffusion-weighted MRI demonstrating no infarction in the bilateral occipital lobes; (F) Axial FLAIR MRI demonstrating bilateral occipital lobe edema; (G) Axial FLAIR MRI demonstrating extension of edema to the left parietal lobe; (H) Axial CT perfusion demonstrating prolonged mean transit time, particularly in the left occipital lobe; (I) CT perfusion demonstrating normal cerebral blood volume, indicating venous stasis. MRI, magnetic resonance imaging; CT, computed tomography; SSS, superior sagittal sinus; TS, transverse sinus; FLAIR, fluid-attenuated inversion recovery

A 34-year-old right-handed female presented with a two-month history of progressive Valsalva-induced headaches. Examination revealed a left partial abducens nerve palsy and left cerebellar syndrome. Neuroimaging demonstrated a large torcular tumor with sinus invasion. Angiogram showed complete obstruction of the torcula, posterior superior sagittal sinus (SSS), and bilateral transverse sinuses (TS), with retrograde venous drainage (Figure 1).

She underwent preoperative Onyx angioembolization (Medtronic, Irvine, CA, USA), followed by gross total resection of the tumor and occluded torcula, SSS, and TS, with preservation of the straight sinus. Immediately postoperatively, the patient developed bilateral severe visual impairment, with only preservation of light perception. There was no evidence of central retinal artery occlusion on ophthalmological assessment, and no acute territorial infarct, intraparenchymal hematoma, or hydrocephalus on neuroimaging; however, computed tomography (CT) venogram and CT perfusion revealed straight sinus thrombosis and associated venous stasis in the bilateral occipital poles, worse on the left than the right. This was corroborated by fluid-attenuated inversion recovery (FLAIR) hyperintensity on magnetic resonance imaging (MRI) (Figures 1F-1G). Biochemical and metabolic screening revealed no abnormalities.

The patient’s visual acuity improved by post-operative day (POD) 4 to counting fingers, but this was accompanied by the onset of complex visual and auditory hallucinations. She reported visions of formed objects, figures, and floating letters/numbers in her right visual field. Notably, she identified these visions as meaningful items from her past, related to her childhood and work. She was also able to hear the voices of the figures, as if they were speaking to her. Electroencephalogram (EEG) showed no epileptiform activity during episodes of visual hallucinations.

Therapeutic subcutaneous Clexane for presumed cerebral venous thrombosis (CVT) had been initiated alongside dexamethasone for vasogenic edema on POD 0. Her symptoms improved over one month, allowing the patient to carry out most daily activities, with mild residual visual disturbances. Visual acuity and fields normalized by 3 months, and repeat CT perfusion also showed normalization of cerebral blood flow (CBF) and mean transit time (MTT). Both visual and auditory hallucinations had fully resolved by six months. Histopathology confirmed it as CNS WHO Grade 1 Solitary Fibrous Tumor [4].

Case 2

**Figure 2:**
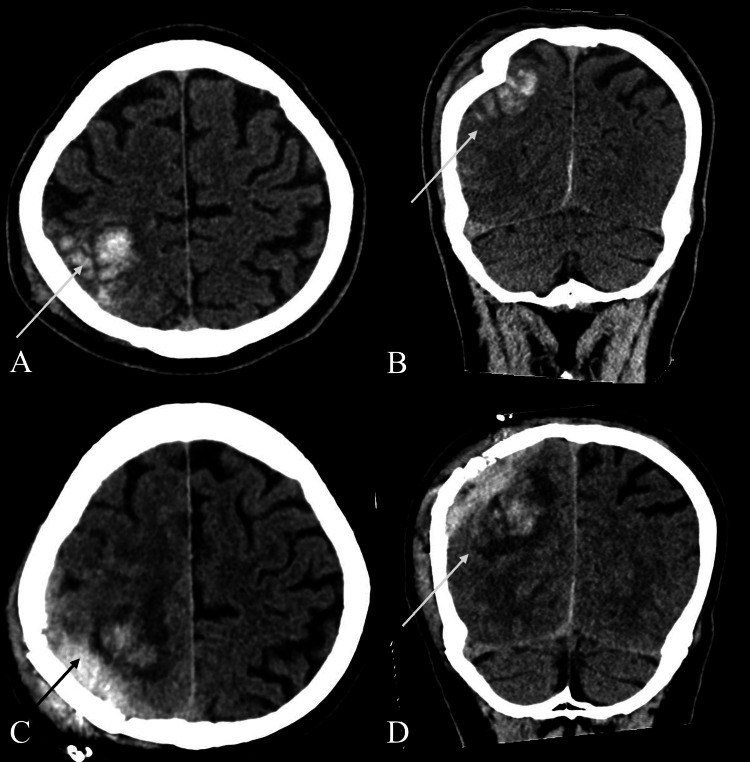
CT Brain Images for Patient 2 (A) Axial view demonstrating right intraparenchymal cortical/subcortical hematomas of the right parietal lobe; (B) Coronal view demonstrating depressed fracture of the right parietal bone, with intraparenchymal hematomas; (C) Axial and (D) Coronal views performed on POD 8, demonstrating increased edema in the right parietal lobe. CT, computed tomography; POD, post-operative day

A 53-year-old right-handed male presented following a fall, with a head injury. Neuroimaging showed a comminuted and depressed right parietal fracture, with associated acute cortical and subcortical intraparenchymal hematomas of the right superior parietal lobule (Figure 2).

He underwent surgical elevation of the depressed skull fracture and was treated with a prophylactic seven-day course of levetiracetam.

The patient was readmitted on day 8 of the head injury after an absence seizure. Imaging showed no hematoma progression, and the patient returned to baseline. The next day, he developed complex left-sided visual and auditory hallucinations, including visions of his workplace and daughter, visualization of numbers 3, 4, and 5, as well as left-ear auditory hallucinations of his daughter’s voice. Examination revealed no focal deficits, abnormal movements, or impaired consciousness. EEG showed no epileptiform activity. No abnormalities were identified on biochemical and metabolic evaluation.

Mannitol was initiated for mild cerebral edema seen on CT, and tapered over a week after improvement of symptoms. His hallucinations resolved completely by day 14 post-injury, with no recurrence at three months’ follow-up.

Systematic review

**Figure 3:**
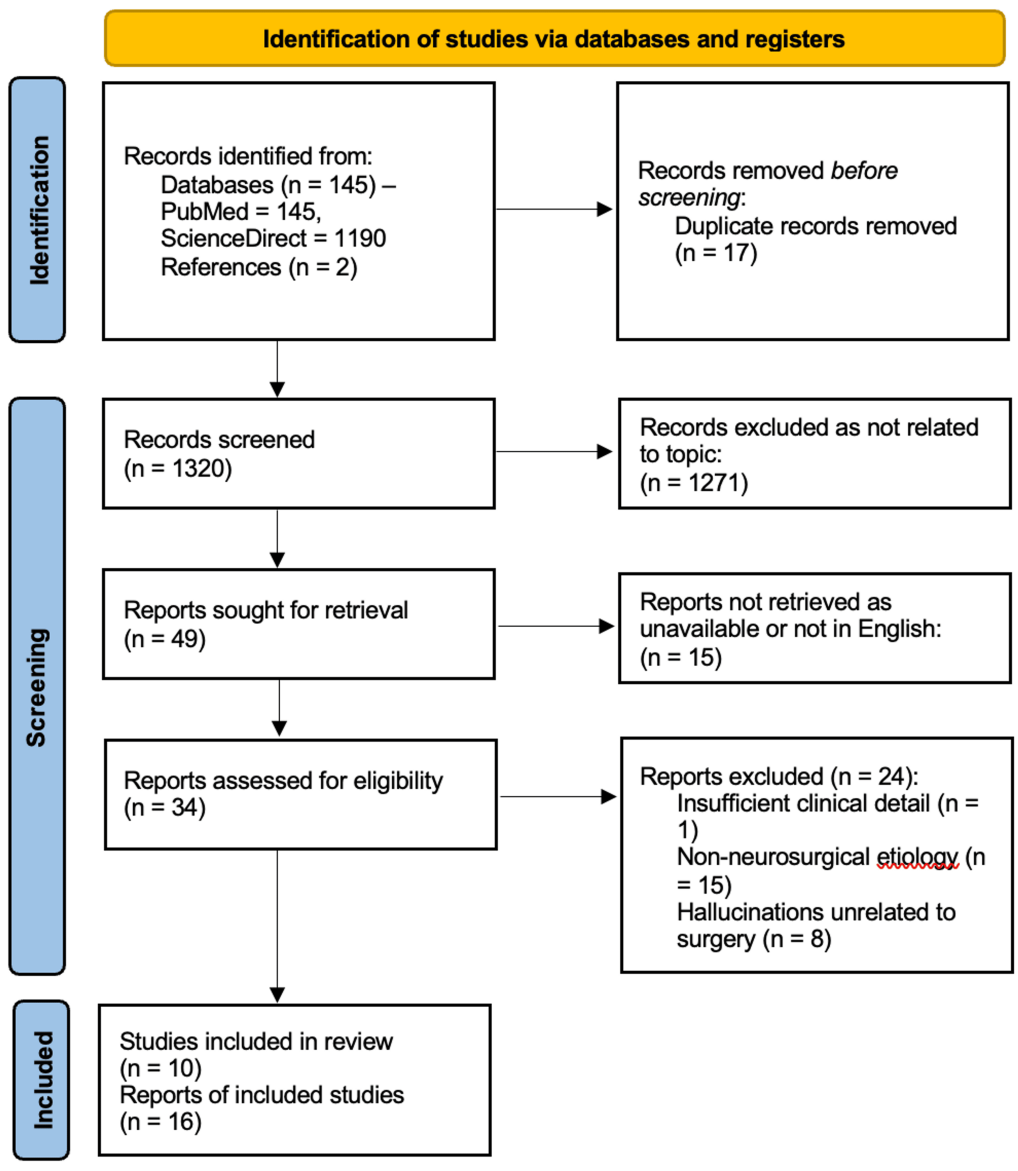
PRISMA Flow Diagram Illustrating Articles Screened and Included in the Qualitative Analysis PRISMA, Preferred Reporting Items for Systematic Reviews and Meta-Analyses

This systematic review was conducted in accordance with the Preferred Reporting Items for Systematic Reviews and Meta-Analyses (PRISMA) 2020 guidelines [5]. A PRISMA flow diagram, outlining the study selection process, is provided in Figure 3.

Records identified through database searching were deduplicated, and two independent reviewers screened titles/abstracts against predefined eligibility criteria. Full-text articles of potentially relevant studies were assessed, with discrepancies resolved via consensus or a third reviewer. The selection process followed the PRISMA flow diagram, documenting: (1) total records retrieved from each database, (2) excluded duplicates, (3) records excluded at title/abstract screening (as not related to topic), (4) full-text articles excluded (with justification, e.g., “non-neurosurgical etiology,” “unrelated to surgery”), and (5) final included studies (case reports/series meeting all criteria).

Protocol and Registration

No formal protocol was registered for this review. However, the review was designed and conducted using PRISMA 2020 standards for transparency and reproducibility.

Eligibility Criteria

Studies were considered eligible for inclusion if they met several criteria. First, the population of interest consisted of neurosurgical patients who had undergone cranial neurosurgical intervention. Second, the outcome had to include documented formed visual and/or auditory hallucinations - defined as structured perceptual experiences, such as images or voices, and not simple flashes, noises, or geometric patterns. Third, eligible study designs included case reports, case series, or observational studies. Finally, only studies written in English and published in peer-reviewed journals were included.

Studies were excluded if hallucinations arose primarily from psychiatric disorders without organic lesions, such as schizophrenia or bipolar disorder. Reports limited to unformed hallucinations, such as flashes of light or geometric visual phenomena, were excluded. In addition, review articles, opinion pieces, animal studies, and abstracts without available full texts were not considered.

Information Sources and Search Strategy

A systematic literature search was performed in PubMed/MEDLINE, from inception to June 12, 2025. The search strategy combined terms for hallucinations and neurosurgical contexts.

For the hallucination component, we combined Medical Subject Headings (MeSH terms) and text-word searches, including: "Hallucinations"[MeSH], "formed hallucination," "complex hallucination," "visual hallucination," and "auditory hallucination." The neurosurgical context was captured using: "Neurosurgical Procedures"[MeSH], "neurosurg*," "craniotomy," and "brain surgery."

The search strategy was adapted for ScienceDirect, while maintaining conceptual consistency across platforms. The search strategy was intentionally broad to capture all relevant case reports. Reference lists of included articles were also screened to identify additional eligible studies.

Selection and Data Collection Process

Two reviewers independently screened all titles and abstracts for relevance. Full-text articles were retrieved for studies meeting the inclusion criteria, or if eligibility was unclear. Discrepancies were resolved by discussion and consensus. A total of 10 studies, reporting 16 individual cases, were included.

Data extraction was conducted independently by two reviewers using a standardized template. Extracted variables included patient demographics such as age; surgical indication (e.g., epilepsy, tumor, and trauma); procedure type and anatomical location; type of hallucination (visual, auditory, or both); timing of symptom onset; frequency and duration of hallucinations; time to symptom resolution; and, when available, histopathology.

Risk of Bias Assessment

Given the nature of the included studies (case reports and case series), formal risk of bias assessment tools (e.g., ROBINS-I [6] or Newcastle-Ottawa Scale [7]) were not applied. Instead, limitations were addressed qualitatively in the Discussion section.

## Discussion

Due to the small number of cases and heterogeneity in the clinical context and reporting, meta-analysis was not feasible. A narrative synthesis was performed, and data were tabulated to identify patterns in symptom onset, anatomical correlates, and resolution timing (Table 1).

**Table 1:** All Reported Cases of Visual and Auditory Hallucinations After Cranial Surgery † denotes current report. Note: Onset refers to the time interval between surgery and the initial appearance of hallucinations. Resolution refers to the complete disappearance of hallucinations, as described by the authors. Frequency/duration is patient-reported when specified; “not described” indicates unavailable detail in the original source.

No.	Author	Age (years)	Surgery	Type/Hallucination Onset	Duration of Occurrence	Time of Resolution (months)
Epilepsy surgery-related						
1	Freiman et al. [8]	40	Amygdalohippocampectomy for epilepsy	Visual/Immediately postoperatively	Daily	4 days
2	Freiman et al. [8]	29	Amygdalohippocampectomy for epilepsy	Visual/After surgery (not described)	Not described	7 days
3	Choi et al. [9]	35	Right occipital cortical dysplasia resection for epilepsy	Visual/2 months postoperatively	Not described	Not described
4	Contardi et al. [10]	49	Left anteromesial temporal lobectomy for epilepsy	Visual/3 days postoperatively	A few seconds to minutes, frequency not described	2
5	Vanags et al. [11]	3	Hemispherectomy for focal seizures (frontal and parieto-occipital disconnection, temporal lobectomy, corpus callosotomy)	Visual/1 month postoperatively	2-3 times/week, 15-20 minutes	3.5
6	Vanags et al. [11]	6	Subtotal hemispherectomy for focal seizures (frontal disconnection, temporal lobectomy)	Visual/18 months postoperatively	1-2 times/week, 20-40 minutes	6
Tumor related						
1	Isolan et al. [12]	34	Resection of right insular glioma	Auditory/1 month postoperatively	Daily, for a few seconds	2
2	Coetzer [13]	Mid-60s	Resection of right frontal meningioma	Auditory/9 months postoperatively	Not described	Not described
3	Maiuri et al. [14]	44	Resection of right cerebellar meningioma (via suboccipital craniotomy)	Visual/1 day postoperatively	Not described	4 days
4	Freiman et al. [8]	38	Resection of left parietal astrocytoma	Visual/After surgery (not described)	Not described	6
5	Freiman et al. [8]	50	Resection of left parieto-occipital glioblastoma	Visual/After surgery (not described)	Not described	21 days
6	Ovchinnikov et al. [15]	74	Resection of left occipital metastases	Visual/Immediately postoperatively	Daily	18
7	Ovchinnikov et al. [15]	76	Resection of left parietal metastases	Visual/1 day postoperatively	Daily	Continued until the patient's demise a few weeks later
8	Grasso et al. [16]	62	Resection of right temporal glioblastoma	Visual/3 days postoperatively	Not described	Present on discharge at 1 month
9	Grasso et al. [16]	77	Resection of left temporal metastases	Visual/3 days postoperatively	Not described	Present on discharge at 10 days
10	Koh et al.^†^	34	Occipital/parietal venous stasis (from torcular tumor resection)	Visual/4 days postoperatively	Daily	6
Trauma						
1	Stewart and Brennan [17]	36	Evacuation of acute extradural and subdural hematoma	Auditory/After surgery (not described)	Not described	6
2	Koh et al.^†^	53	Right parietal edema from worsening contusion	Visual/8 days postoperatively	Daily	0.5

The reviewed cases demonstrate considerable variability in hallucination onset and duration. Symptom emergence ranged from immediate postoperative periods to delayed presentations at 18 months post-procedure, with epilepsy-related cases typically manifesting earliest (immediate to three days postoperatively). The majority of cases (92%) resolved within six months, though intra-axial lesions - particularly tumor-related cases - were associated with more prolonged symptoms, persisting up to 18 months.

Visual hallucinations predominated (80% of cases), potentially reflecting both the greater vulnerability of visual processing networks to surgical manipulation and enhanced clinical recognition of visual phenomena. Auditory hallucinations, while less common (20%), were exclusively associated with temporal lobe and insular procedures, consistent with known auditory processing pathways.

Hallucinations in neurosurgical patients are rarely reported and represent a diagnostically challenging, yet clinically significant, phenomenon that warrants careful consideration. We contribute two cases of complex formed visual and auditory hallucinations following distinct neurosurgical pathologies to the growing literature: (1) parieto-occipital lobe venous stasis from sinus thrombosis following torcular tumor resection, and (2) progressive parieto-occipital edema due to superior parietal lobule contusion, and we completed a systematic review describing its clinical course and prognosis.

Systematic review

Our systematic review identified only 16 documented cases of complex visual and auditory hallucinations occurring after neurosurgical procedures, across diverse etiologies including epilepsy surgery, tumor resection, and traumatic brain injury. This was surprisingly rare, given the frequency of cranial procedures. Several notable patterns emerged.

Complex visual hallucinations were the most frequently reported type (81% of cases), while isolated auditory hallucinations were comparatively less common and typically linked to intra-axial lesions or interventions involving the temporal or insular regions - consistent with known auditory processing pathways. Notably, to our knowledge, no published neurosurgical literature has described concurrent visual and auditory hallucinations in the same patient. These dual-modality hallucinations are predominantly described in psychiatric contexts, raising the question of whether neurosurgical patients with both types of hallucinations are under-recognized or underreported.

Secondly, most visual hallucinations were associated with the identified pathology involving the parieto-occipital region, suggesting a regional predilection. This aligns with existing literature implicating the lateral occipital cortex and temporo-parietal junction in formed visual hallucinations [18], whereas unformed hallucinations (e.g., flashes and lights) tend to localize medially within the primary visual cortex in the calcarine sulcus [19]. The predominance of intra-axial tumor-related lesions also suggests the possibility that white matter disruption, rather than direct cortical injury, may be an important contributor in the pathophysiology of hallucinations. In addition, auditory hallucinations in our patients, with parieto-occipital involvement without temporal or insular dysfunction, further underscore this hypothesis of white matter tract disruption.

No definitive relationship was identified between the lesion location (intra- versus extra-axial) and hallucination characteristics in neurosurgical patients, due to limited case numbers. Hallucinations related to epilepsy surgery were observed to exhibit earlier onset and more prolonged symptoms, likely reflecting distinct pathophysiological mechanisms due to chronic epileptogenic networks, residual epileptogenic activity, or ictal phenomena. These should be interpreted separately from hallucinations arising after tumor or traumatic brain injury surgery, where structural disruption and perilesional edema are likely to play a more prominent role.

Our two cases notably present simultaneous, vividly formed visual [20] and auditory hallucinations, localized to the parieto-occipital association cortex - an uncommon and previously unreported phenomenon in neurosurgical patients. This pattern suggests transient disruption of multimodal integration networks, rather than focal sensory disturbance. EEG findings were negative for epileptiform activity, further supporting a non-epileptic etiology. In Case 2, symptoms resolved completely within one month, consistent with reversible cerebral edema. In Case 1, improvement occurred more gradually, paralleling the development of collateral venous drainage and resolution of venous congestion.

Clinical implications

Both patients manifested vivid, meaningful visual and auditory hallucinations without accompanying seizure activity, supporting non-epileptogenic mechanisms and network-level dysfunction, instead of localized cortical pathology [21,22]. These observations highlight several key implications: the need for clinical suspicion and exclusion of seizure activity, the value of tailored, etiology-specific management, and the typically favorable prognosis for complete symptom resolution with timely intervention.

A major diagnostic challenge in recognizing hallucinations within neurosurgical practice stems from a characteristically low index of suspicion. These phenomena are frequently misattributed to more common postoperative complications, such as delirium, metabolic disturbances, or medication side effects [23,24]. Furthermore, as illustrated in the systematic review (Table 1), the variable presentation timeline and sensory modalities complicate recognition. The first case highlights this diagnostic pitfall, where the patient's complex visual hallucinations following tumor resection were initially concerning for posterior circulation ischemia, before advanced imaging revealed the true underlying pathophysiology of venous sinus thrombosis and occipital lobe edema. Similarly, the second case's post-traumatic hallucinations were initially suspected to represent postictal phenomena, yet comprehensive evaluation, including EEG and serial neuroimaging, confirmed their origin in traumatic subcortical injury without epileptiform activity. Failure to recognize this difference would have labeled the patient with epilepsy, which may have negative implications for the patient’s ability to drive and employment.

This diagnostic process highlights the need to maintain a broad differential in neurosurgical patients with new-onset perceptual disturbances, warranting systematic evaluation with biochemical and metabolic tests, structural and functional imaging, vascular studies, and electrophysiological monitoring, to identify and exclude treatable causes.

The therapeutic importance of treating the underlying etiology emerges as a consistent theme in both cases. In the first patient, anticoagulation for venous sinus thrombosis, coupled with corticosteroid therapy for vasogenic edema, could have possibly resulted in accelerated symptomatic and radiological resolution. Addressing the issue early may also have prevented thrombus propagation, eventually tipping the patient over into malignant venous hypertension. This clinical course supports the hypothesis of venous hypertension and cortical dysfunction in the pathogenesis of visual hallucinations [25,26]. In the second case, treatment of post-traumatic edema led to symptom resolution, reinforcing the principle that effective treatment of hallucinations necessitates correction of the precipitating structural or physiological disturbance. These observations align with growing evidence that hallucinations reflect downstream manifestations of primary neural network disruption [27], and that symptom resolution is most reliably achieved by addressing the underlying pathology rather than focusing solely on symptomatic suppression with antipsychotics.

The favorable prognosis of hallucinations in our cases is reflected by the underlying etiology of edema. This suggests that when perceptual disturbances result from temporary network dysfunction, instead of permanent structural damage, intrinsic neuroplasticity may facilitate recovery. This has implications for patient counseling, prognostication, and therapeutic decision-making. Other etiologies, such as scar and direct injury of implicated white matter tracts, may not have similarly good recovery.

Limitations

This study has several limitations. First, the small number of cases in both our series and the existing literature limits the generalizability of findings and precludes definitive conclusions regarding causality or anatomical correlations. Second, the retrospective nature of the case reports and literature review introduces inherent bias and limits standardization in data collection. Third, clinical documentation was often incomplete or inconsistent, particularly regarding the qualitative nature of hallucinations. In many cases, there was no clear distinction between formed and unformed phenomena, whether hallucinations were memory-based or random, or their duration and frequency - limiting deeper pathophysiological insights. Standardized prospective data collection and use of validated phenomenological tools would be valuable in future studies.

## Conclusions

Complex visual and auditory hallucinations are rarely reported in neurosurgical patients. These two described cases, secondary to transient disturbances of the parieto-occipital lobe, highlight the potential role of association cortices and underlying white matter tracts implicated in multisensory perception. Exclusion of seizure activity is essential in evaluation, and prognosis appears to be more favorable in reversible, non-structural conditions.
